# Delta III reverse shoulder arthroplasty in the treatment of complex 3- and 4-part fractures of the proximal humerus: 6 to 42 months of follow up

**DOI:** 10.1186/1471-2474-14-231

**Published:** 2013-08-08

**Authors:** Georg Mattiassich, Lucian Lior Marcovici, Rolf Michael Krifter, Reinhold Ortmaier, Peter Wegerer, Albert Kroepfl

**Affiliations:** 1 Trauma Center Unfallkrankenhaus Linz, Teaching Hospital of the Paracelsus Medical University Salzburg, Garnisonstrasse 74017, Linz, Austria; 2 Department of Orthopaedic and Trauma Surgery, “Sapienza” University of Rome, Piazzale Aldo Moro 500185, Rome, Italy; 3 General and Orthopedic Hospital LKH Stolzalpe, Teaching hospital of Graz Medical University, Stolzalpe 388852, Stolzalpe, Austria; 4 Department of Trauma Surgery, Paracelsus Medical University and Salzburger Landeskliniken, Müllner Hauptstraße 485020, Salzburg, Austria

**Keywords:** Reverse shoulder arthroplasty, Proximal humerus fracture, Joint prosthesis

## Abstract

**Background:**

There is a growing tendency for complex proximal humerus fractures (PHF) in osteoporotic patients to be treated with reverse shoulder arthroplasty (RSA). It has been proposed that RSA has more benefits than other treatment options. The aim of our study was to investigate preoperative characteristics as well as clinical and radiological outcomes in patients with complex 3- or 4-part PHF who had undergone primary RSA.

**Methods:**

Patients with a minimum follow-up of 6 months who had undergone a primary RSA after 3- or 4-part PHF in the period between 2008 and 2011 were eligible for the study. Clinical records, X-rays and CT-scans were investigated and a clinical examination was performed. Disabilities of the Arm, Shoulder and Hand (DASH) score and Constant-Murley score (CMS) were calculated. Sixteen patients were examined as part of the study. The mean follow-up was 20 months (range 6-42 months). According to Codman-Hertel classification we encountered 15 Hertel “12” and 1 Hertel “8” type fractures.

**Results:**

Thirty-two patients (27 female – 84.4%) with a mean age of 72 years underwent operations to treat complex 3- and 4-part fractures of the proximal humerus. Sixteen patients were reexamined. In 14 cases the dominant upper extremity was on the right, in 2 cases it was on the left, in 6 cases the right side was affected and in 10 cases the left side was affected. The mean CMS was 54.8 (range 18-95) and the mean DASH was 37.5 (range 2.9-81). A trend was established between the CMS and dominance of the affected shoulder. The CMS was better if the affected shoulder was on the non-dominant side (p-value 0.051). No statistical difference was noted between age and clinical outcome.

**Conclusions:**

Our mid-term follow-up shows satisfying results in terms of the treatment of severe displaced fractures in elderly patients with RSA. RSA can provide immediate relief and good shoulder function in elderly patients. Nevertheless, the question of longevity of these implants remains to be observed.

## Background

Vertebral fractures, hip fractures, distal forearm fractures and humeral fractures are the most common osteoporotic fractures. The average lifetime risk in a 50 year old Caucasian of experiencing a humeral fracture has been estimated at 12.9% for women and at 4.1% for men [1].

There is increasing incidence of such fractures due to the increasing mean age of the population and the higher levels of activity among the elderly. Palvanen et al. showed that in the over 60 age group in Finland the incidence of proximal humerus fractures tripled between 1970 and 1998 and they anticipate that this trend will continue until 2030 [2].

Possibilities for treating for such fractures range from conservative treatment to operative options such as the plate or nail fixation, humerus-block or other k-wire based systems, hemiarthroplasty or the reversed shoulder arthroplasty (RSA) [3-5].

Grammont invented the RSA for rotator cuff tear arthropathies in 1985 [6]. The indication for PHF in the elderly as an alternative to hemiarthroplasty and plate fixation has broadened in recent years as the number of complications associated with osteosynthesis with unfavourable functional outcome is high in this group of patients due to osteoporotic bone structure as well as the high risk of avascular necrosis of the humeral head and the lack of sufficient rotator cuff [7].

There is a growing tendency for complex PHF in osteoporotic patients to be treated with RSA. It has been proved that patients treated with RSA are easier to mobilize, require less time in hospital and have a better functional outcome after 6 months compared with patients who have undergone other forms of treatment [8].

The unsolved questions of RSA are the need of refixation of the tubercles, the lack of retroversion and the lack of further solutions in case of failure, as we do not have many long-term results.

We set out to investigate preoperative characteristics as well as clinical and radiological outcomes in patients who had undergone a primary RSA as treatment for complex 3 or 4-part PHF in our department in the period from January 2008 to December 2011.

## Methods

### Patients

During the study period from January 2008 and December 2011, 32 patients (27 female – 84.4%) underwent a primary RSA for proximal humerus fracture. The study was performed with the approval of the Institutional Review Board of the AUVA - Austrian social insurance for occupational risks. All patients were contacted in accordance with guidelines set up by the Committee and signed a consent form after being fully informed about the study.

#### Clinical records and investigation

All available X-rays and computer tomography scans (CT-scans) were analyzed with regard to the type of fracture, the direction of dislocation, the length of posteromedial hinge and axis of dislocation. The fractures were classified according to the Neer-, the AO- and the Codman-Hertel classifications.

Clinical records were reviewed for information on patient demographics, co-morbidities, course of trauma, affected side, hand dominance and time to operation. Operation reports were analyzed with regard to duration of procedure, type and size of used prosthesis and the refixation of the tubercles. Rehabilitation protocols were evaluated on the start of active motion.

#### Positioning and procedure

All patients were positioned in the up-right beach chair position. All patients underwent the procedure using the cemented Delta Xtend™ (DePuy-Johnson&Johnson, Warsaw, IN) Reverse Shoulder System. Three highly experienced shoulder fellowship trained surgeons implanted all of the RSAs.

In all cases a Delta-split or deltopectoral approach was used. The approach depended on the direction of the dislocation, the type of fracture and the position of the dislocated head. Tuberosity refixation was attempted in all cases using non-absorbable sutures, which were fixed to the remaining humeral bone or where this was not possible without tension then to holes in the stem.

#### Postoperative phase

Patients were postoperatively immobilized with a Gilchrist-bandage, which remained in place for 4 to 6 weeks after the operation. Start of active assisted motion was defined by the primary surgeon and depending on intraoperative stability began from the 2nd to the 10th day post operation. Our physiotherapists mobilized the patients’ shoulders. The patients were regularly examined in our outpatient clinic. A rehabilitation program under medical surveillance was continued for several weeks until maximum recovery was attained.

#### Protocol

In the period between September and December 2011, 16 patients were available and were examined clinically and radiologically for study purposes. The radiological reassessment included X-ray of both shoulders in antero-posterior (AP) and Y-view. The images were analyzed and focused on loosening of the prosthesis, healing of tubercles and notching. Partial resorption of the tubercle was defined as loosening of 25 to 50% in the initial height of the tubercle and full resorption defined as more than 50% of initial height.

The clinical examination included measurement of the range of motion of both shoulders, the grip strength of both hands measured using the Jamar dynamometer. Pain experienced in motion and under load was assessed using the visual analogue scale (VAS).

The Disabilities of the Arm, Shoulder and Hand (DASH) score and the Constant-Murley score (CMS) forms were completed. Upon completion of the radiological and clinical examination the parameters were recorded in the case report form and computerized for statistical analysis.

#### Statistical analysis

Quantitative data is given as “minimum”, “maximum”, “range”, “arithmetic mean” and “standard deviation”. Correlations were calculated using the Pearson Correlation coefficient. The Kruskal-Wallis one-way analysis of variance and Mann Whitney U test were used for non-parametric testing. Systat 12 (Systat Software, Inc., Chicago, IL) was used to perform statistical calculations.

#### Follow-up study

Sixteen patients were excluded from the study (7 due to follow up of less than 6 months, 4 did not respond, 2 due to dementia, 1 died, 1 had an explantation of the prosthesis after infection, 1 declined to participate in the study). The mean age of the study population at trauma was 72.0 years (range from 60 to 89 years). The mean body mass index (BMI) was 28.9 kg/m^2^ (Table 1). The mean follow up was 20 months (median 17 months, range 6-42 months). An overview of results and other characteristics of patients observed in the follow up study is provided in Table 2.

**Table 1:** Characteristics of study patients

**Details**	**Patients**
Patients	32
Women/Man (%)	27(84.4%)/5(15.6%)
Excluded From The Study	16 patients
Follow up < 6 month	7 patients
Did not respond on call	2 patients
Dementia	2 patients
Decease	1 patient
Prosthesis explanation	1 patient
Refused entering the study	1 patient
Entered In The study	16 patients
Mean Age (range)	72 years (60-89 years)
Mean BMI (range)	28.9 kg/m^2^
Mean Follow up (range)	20 months (6-42 months)

**Table 2:** Results and other characteristics of patients

	**Age at trauma**	**Age at follow up**	**DASH**	**Constant-Murley-score**	**Time operation (min)**	**Months treatment**	**Follow-up**	**Deficit abduction**
N of Cases	16	16	16	16	16	16	16	16
Minimum	60	63	2,9	18	80	1	6	0
Maximum	89	90	81	95	142	14	42	100
Range	29	27	78,1	77	62	13	37	100
Arithmetic Mean	72	73,75	37,5	54,75	108	5,2	19,8	37,5
Standard Deviation	8	7,6	24	19,1	18,6	3,8	11,9	34,7

The cause of trauma was a fall of less than 2 meters in 11 cases, a fall above 2 meters in one patient, 2 high velocity traffic accidents (at more than 50 km/h) and 1 low velocity traffic accident. Ten of the 16 patients were affected on their dominant side. The time to operation was 8.5 days (median of 7, range 0 – 26 days). Three patients were operated on after more than 20 days because patients refused to undergo an RSA as recommended by their surgeon. The other patients were operated on within the first 9 days.

**Figure 1:**
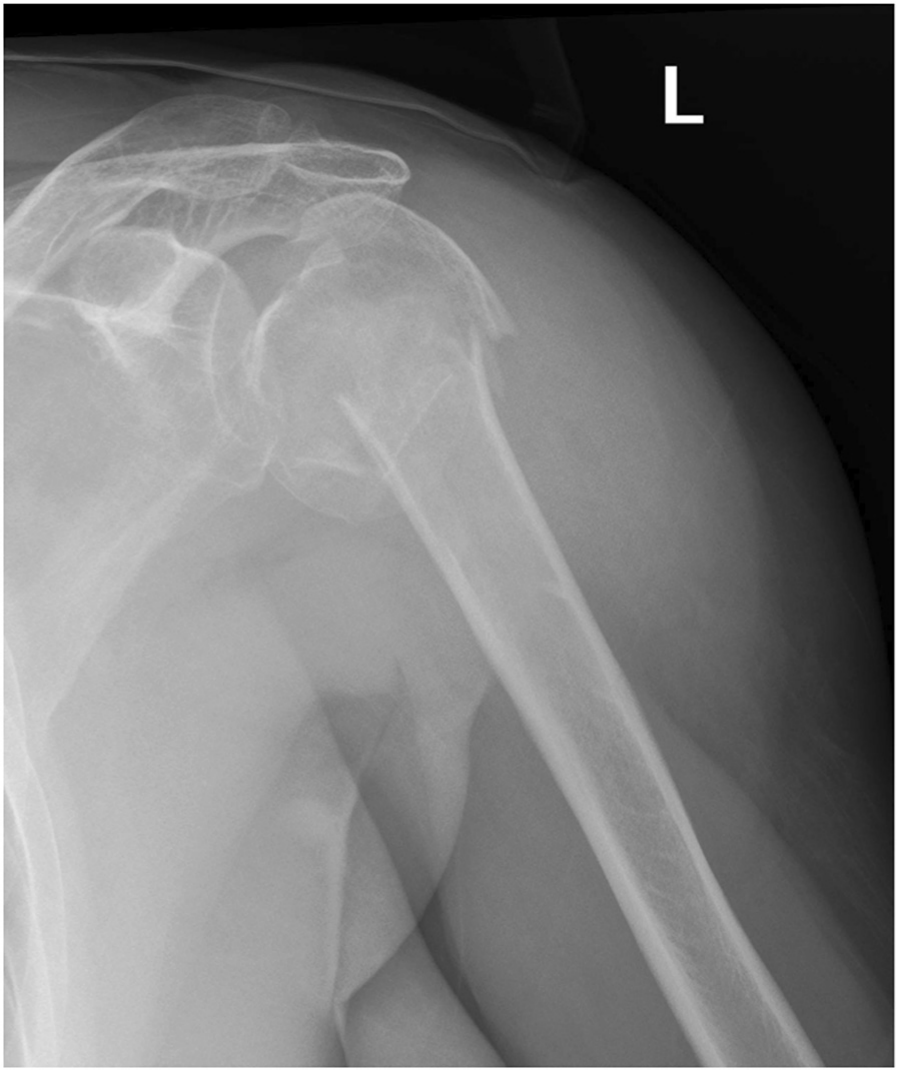
X-ray after trauma (a.-p.-view).

**Figure 2:**
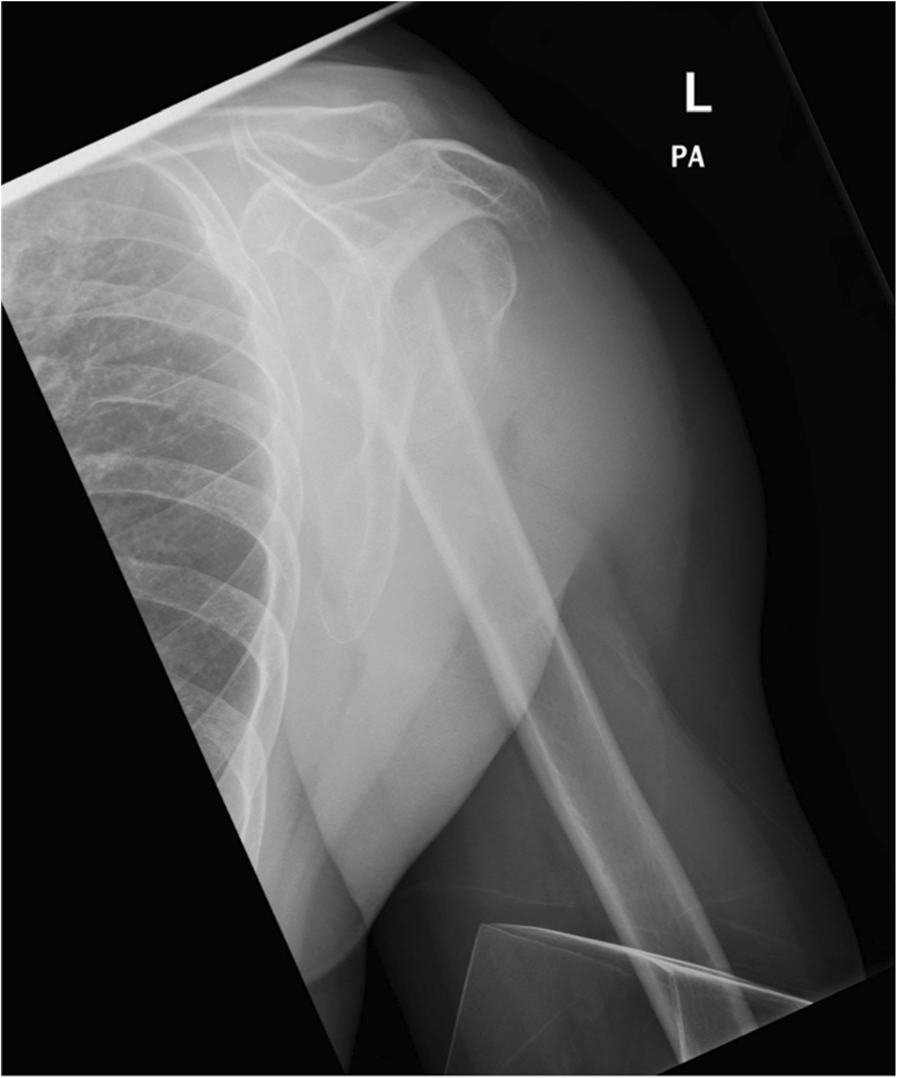
X-ray after trauma (Y-view).

**Figure 3:**
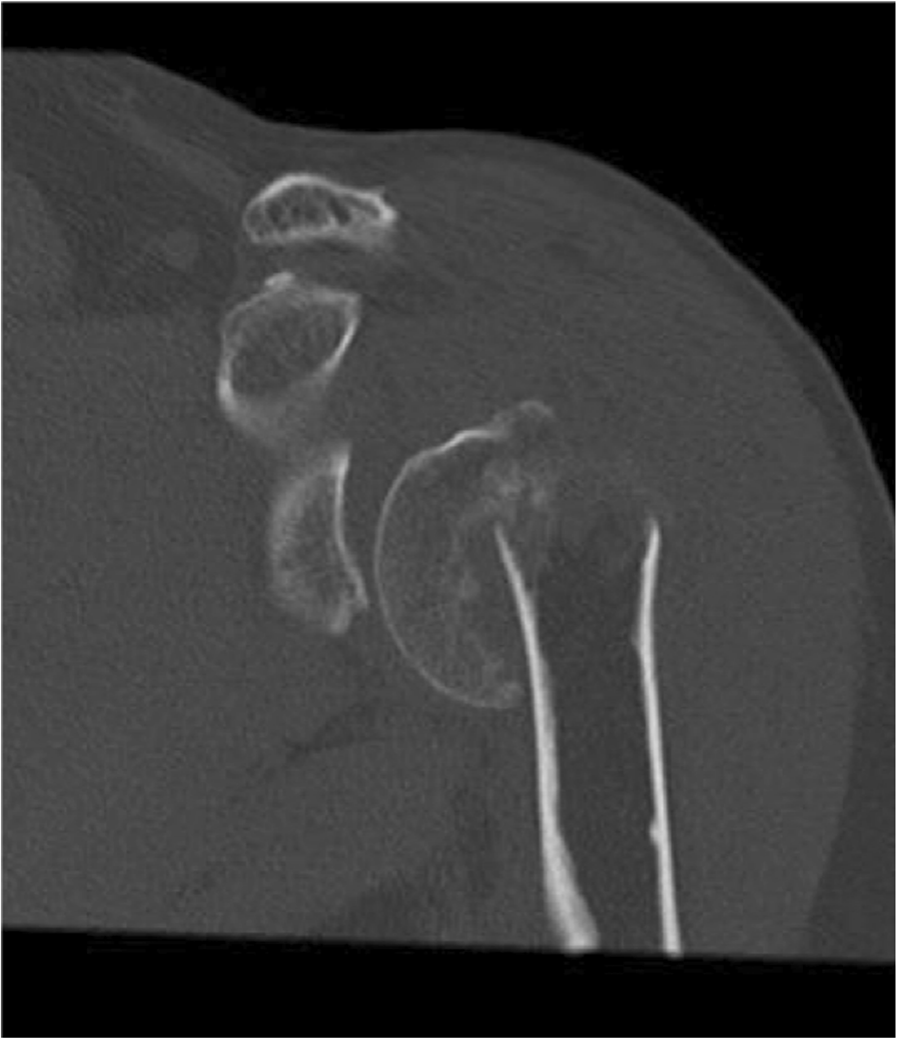
CT-scan after trauma.

All fractures were attributed to C-type according to AO-classification. According to Neer classification, 15 4-part fractures and 1 3-part fracture were encountered. According to Codman-Hertel classification, we encountered 15 Hertel “12” and 1 Hertel “8” type fractures (Figures 1, 2, 3). Eight fractures were dislocated anteriorly and 8 posteriorly. 14 had a posteromedial metaphyseal hinge greater than 8 mm. Seven humeral heads were dislocated in valgus and in 3 cases the head presented splitting of more than 20%.

**Figure 4:**
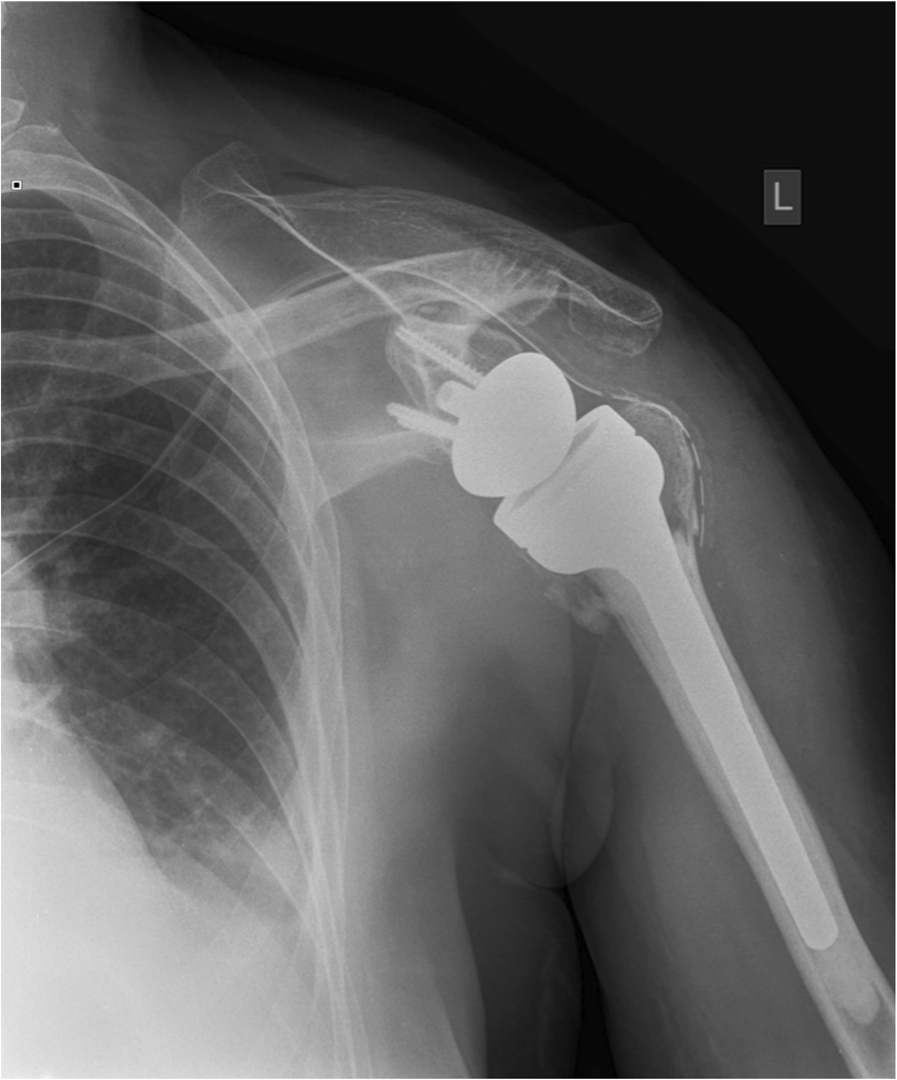
X-ray after implantation of reverse shoulder arthroplasty with refixation of greater tuberosity (a.-p.-view).

**Figure 5:**
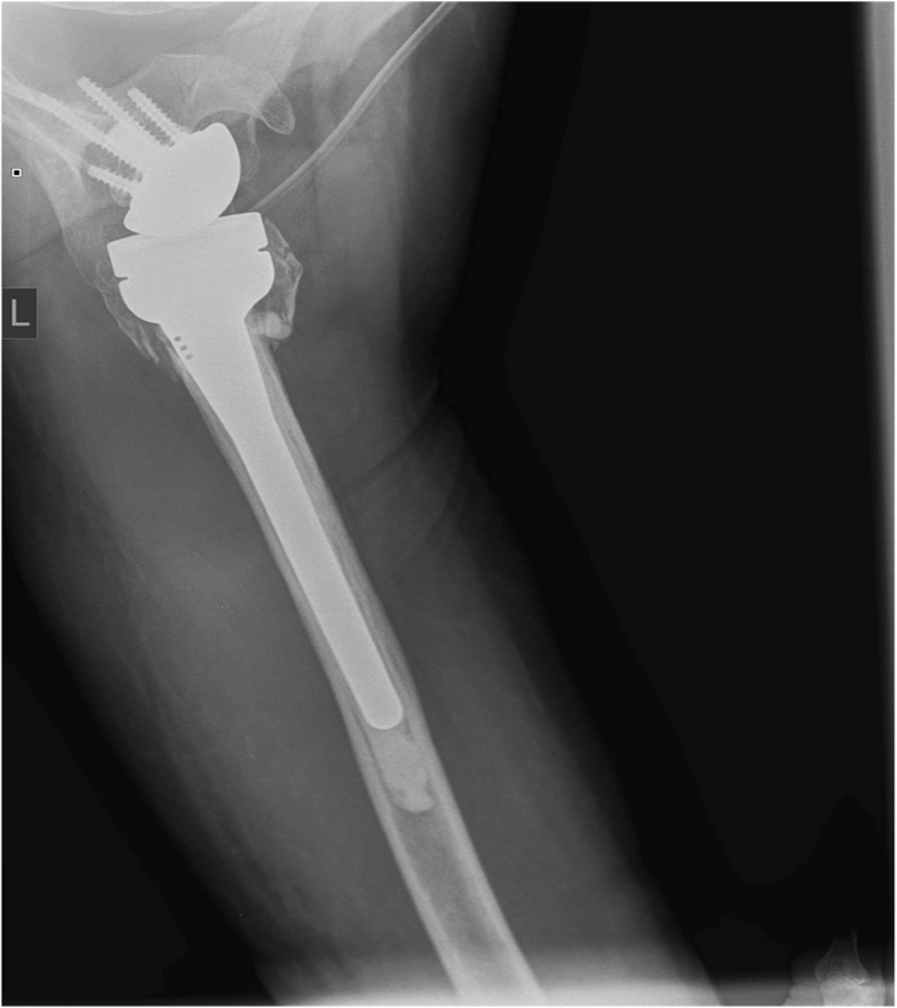
X-ray after implantation of reverse shoulder arthroplasty (axial-view).

The deltopectoral approach was chosen in 2 cases due to the proximity of the humeral head posteriorly to the subscapularis muscle. A well-established cemented reverse monobloc prosthesis (Delta Xtend™ (DePuy-Johnson&Johnson, Warsaw, IN) was used in all cases (Figures 4 and 5). Retroversion was set to 0°. Four screws were used for the fixation of the metaglene. The metaglene was positioned as low as possible and tilted slightly downwards. The size of the glenosphere was between 38 and 42.

Strong and stable refixation of the greater and lesser tuberosity was attempted in all cases using strong sutures around the neck of the prosthesis (Figures 4 and 5). Bone grafting was not needed in any of the patients. The mean operation time was 108 minutes (median 107 min, range 80 to 142 min).

Mean hospitalization time was 19 days (range 10-38 days). Three of the patients stayed in hospital for more than 35 days. This was due to a complex clinical situation as they were severe polytrauma patients. The mean time between the operation and the start of active movement was 12.5 days (range 1-28 days). 4 patients had started active movement after more than 15 days due to their compromised clinical situation.

## Results

### Complications

We experienced one case of transient axillary nerve impairment, one case of superficial wound infection, one deep infection that underwent revision surgery, one dislocation which also underwent revision surgery with a thicker inlay and one patient presented persisting pain resistant to medical therapy. This patient suffers from major depression.

#### Clinical results

**Figure 6:**
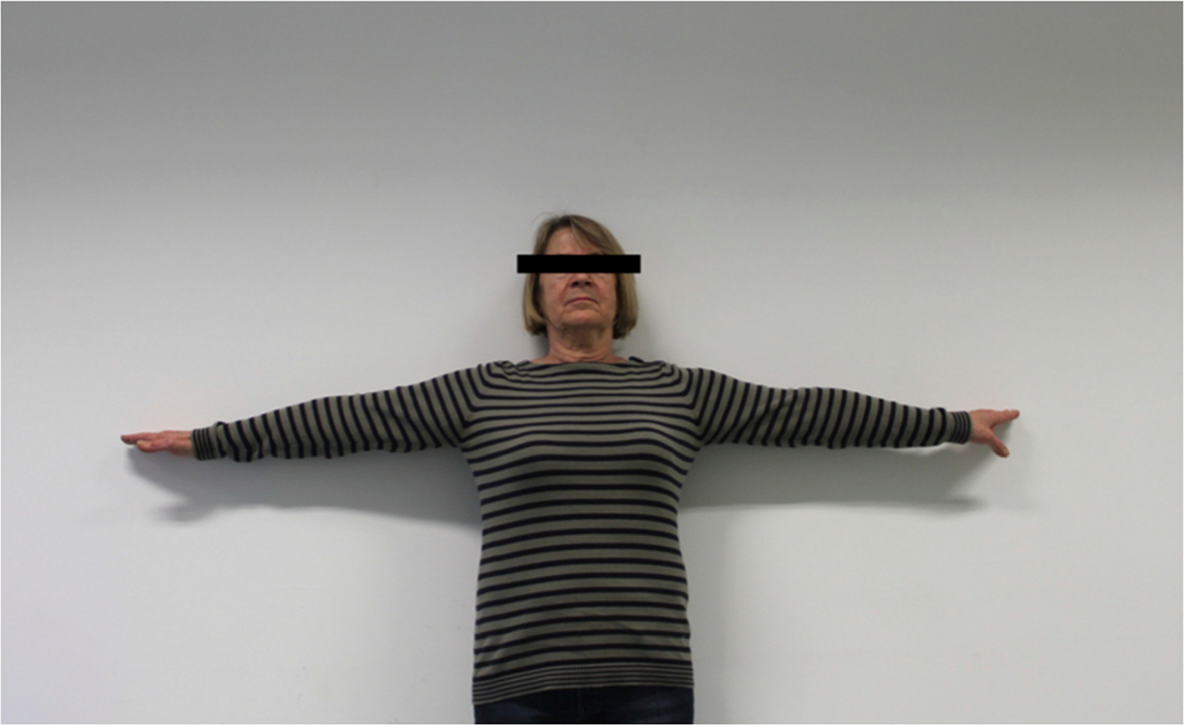
Clinical outcome with satisfying result after one year in abduction (affected shoulder left side).

**Figure 7:**
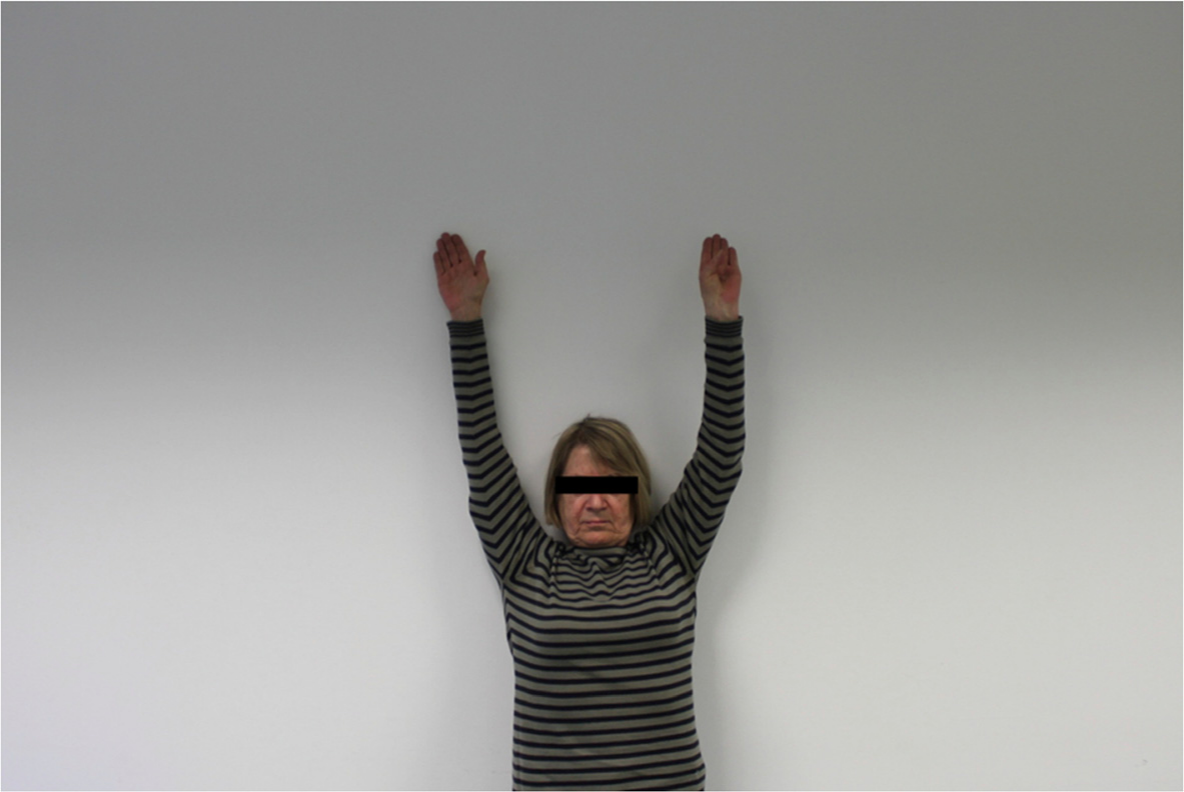
Clinical outcome with satisfying result after one year in maximum abduction (affected shoulder left side).

**Figure 8:**
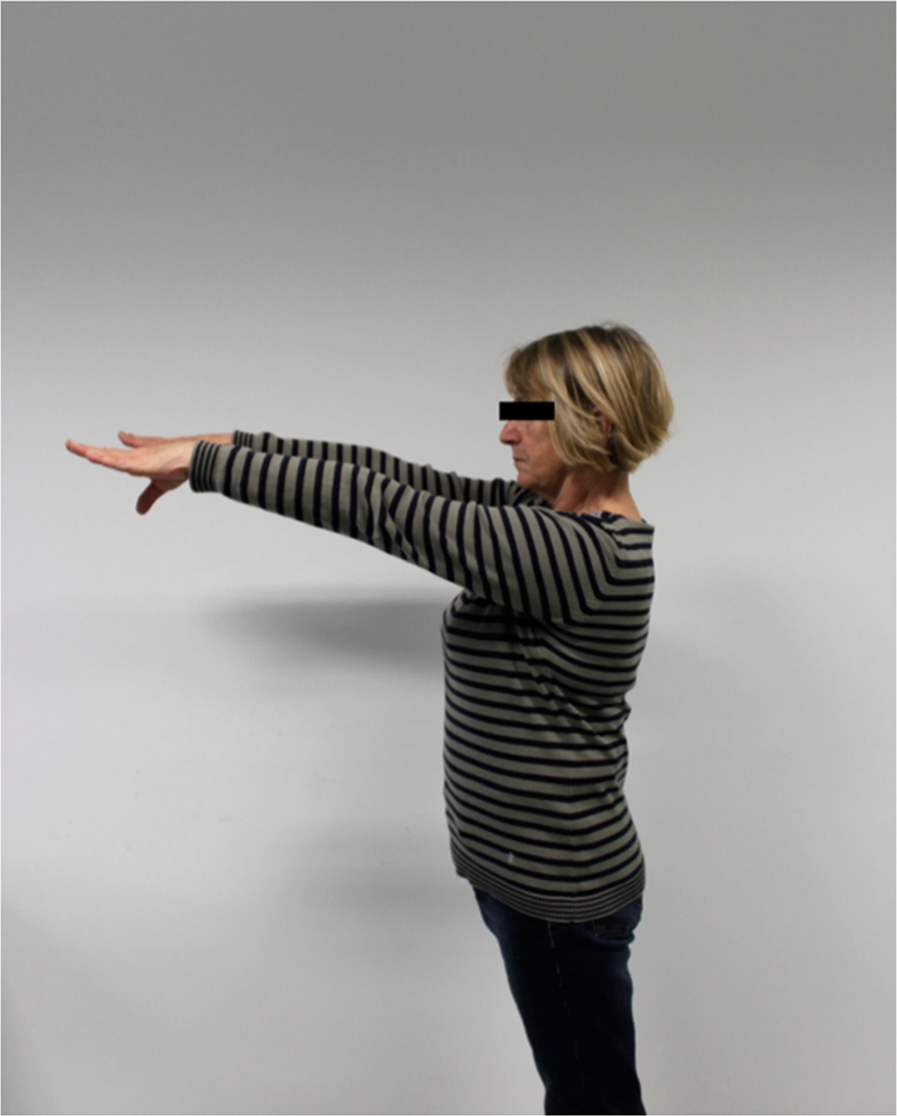
Clinical Clinical outcome with satisfying result after one year in flexion (affected shoulder left side).

**Figure 9:**
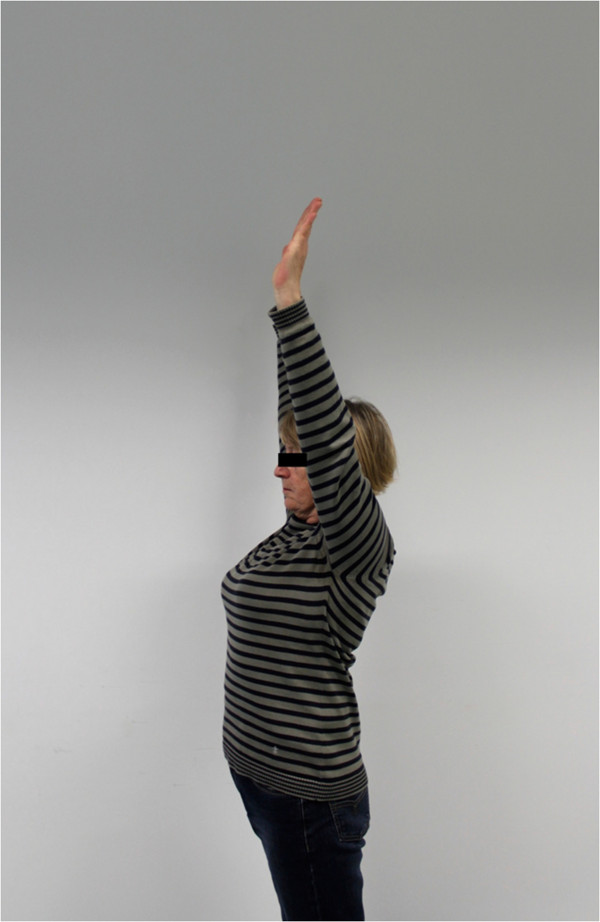
Clinical outcome with satisfying result after one year in maximum flexion (affected shoulder left side).

**Figure 10:**
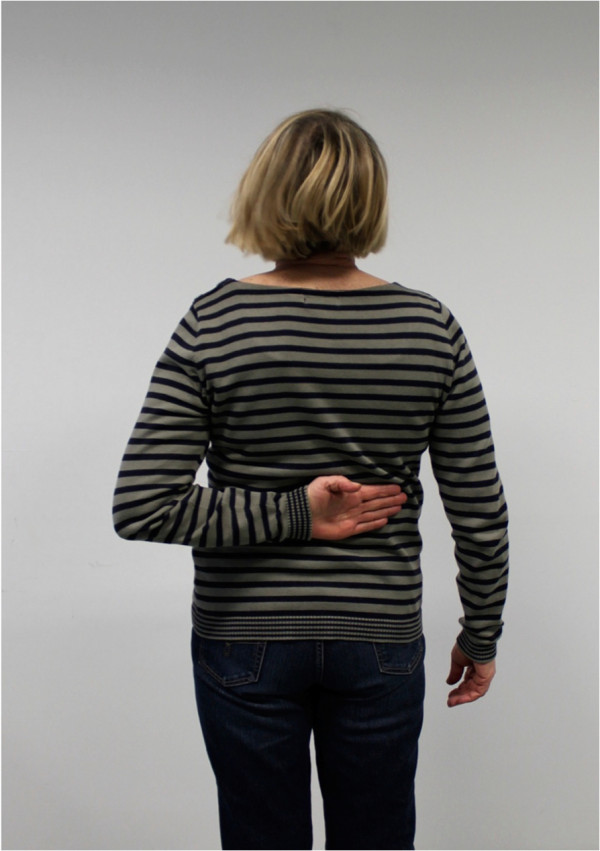
Clinical outcome with satisfying result after one year in internal rotation (affected shoulder).

**Figure 11:**
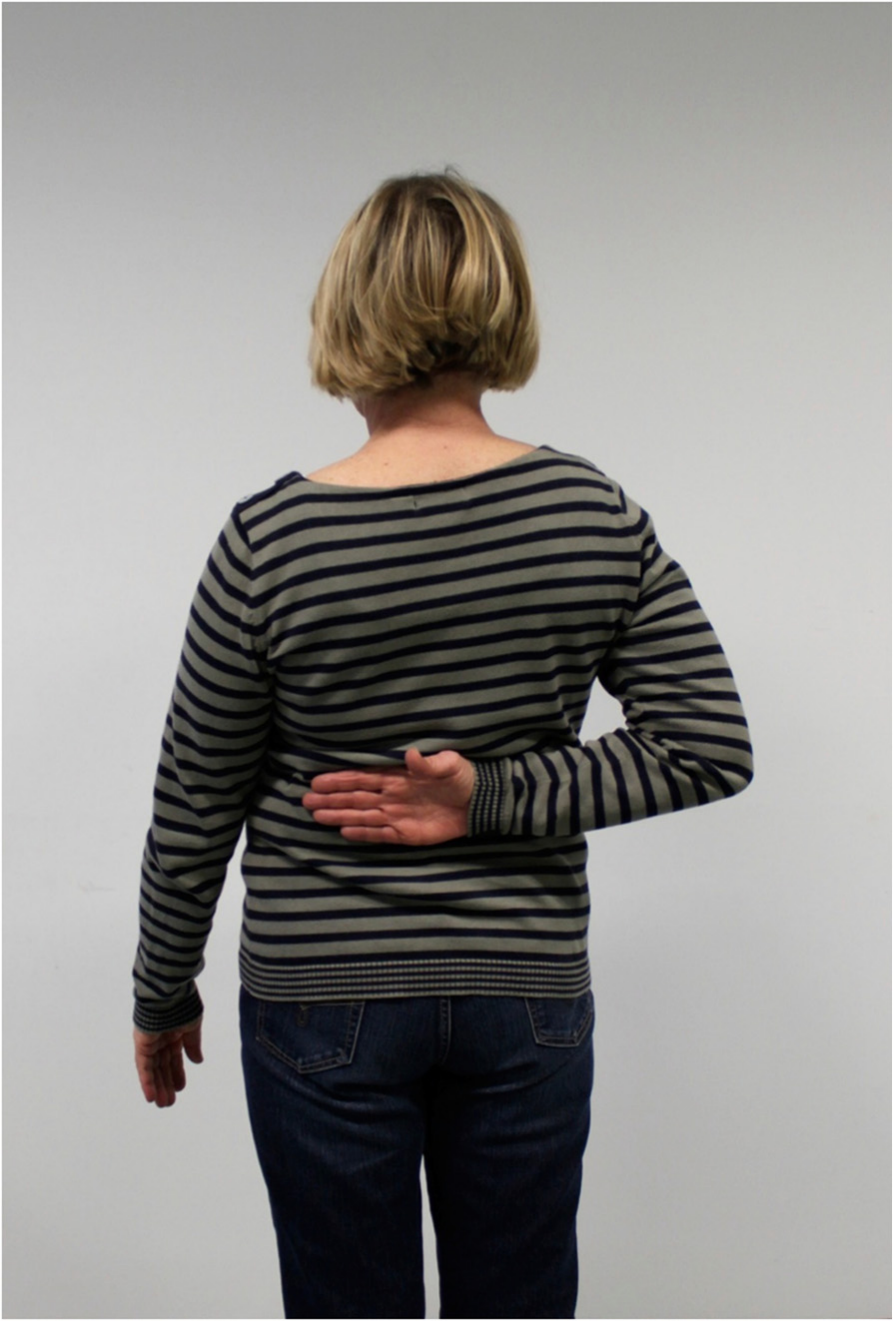
Clinical outcome with satisfying result after one year in flexion (non-affected shoulder).

The postoperative mean range of motion at follow up was: abduction 106.9° (50°-180°); flexion 115.6° (50°-170°); extension 36.3° (20°-60°); external rotation 20.6° (0°-50° internal rotation 50.3° (30° to 70°) (Figures 6, 7, 8, 9, 10, 11). The grip strength measured using the Jamar dynamometer on the affected side was: 80.3 mmHg (30-180 mmHg). The Visual Analog Score (VAS) was: 2.5 (0-8).

The CMS was 54.8 (range 18-95), the DASH was 37.5 (range 2.9-81). There is correlation between the two scores (Pearson correlation -0.879). However, less correlation was observed between age, abduction deficits and the two scores (Table 3).

**Table 3:** Correlation between DASH, Constant-Murley-score and abduction deficit

	**Age at follow up**	**DASH**	**CMS**	**Abduction deficit**
Age at follow up	1,000			
DASH	-0,386	1,000		
CMS	0,168	-0,879	1,000	
Abduction deficit	-0,048	0,484	-0,627	1,000

A trend was established between the CMS and the dominance of the affected shoulder. The CMS was better if the affected shoulder was on the non-dominant side (p-value 0.051 – Table 4). No significant statistical difference was noted between age and clinical outcome. We encountered notching grade I in 7 patients. There were no signs of notching in 9 patients. We encountered no acromion fractures and no non-unions at the fracture site.

**Table 4:** Correlation between CMS and affection of non-dominant shoulder

		
Mann-Whitney U test statistic	:	48,000
p-value	:	0,051
Chi-square Approximation	:	3,817
df	:	1

#### Radiological results

**Figure 12:**
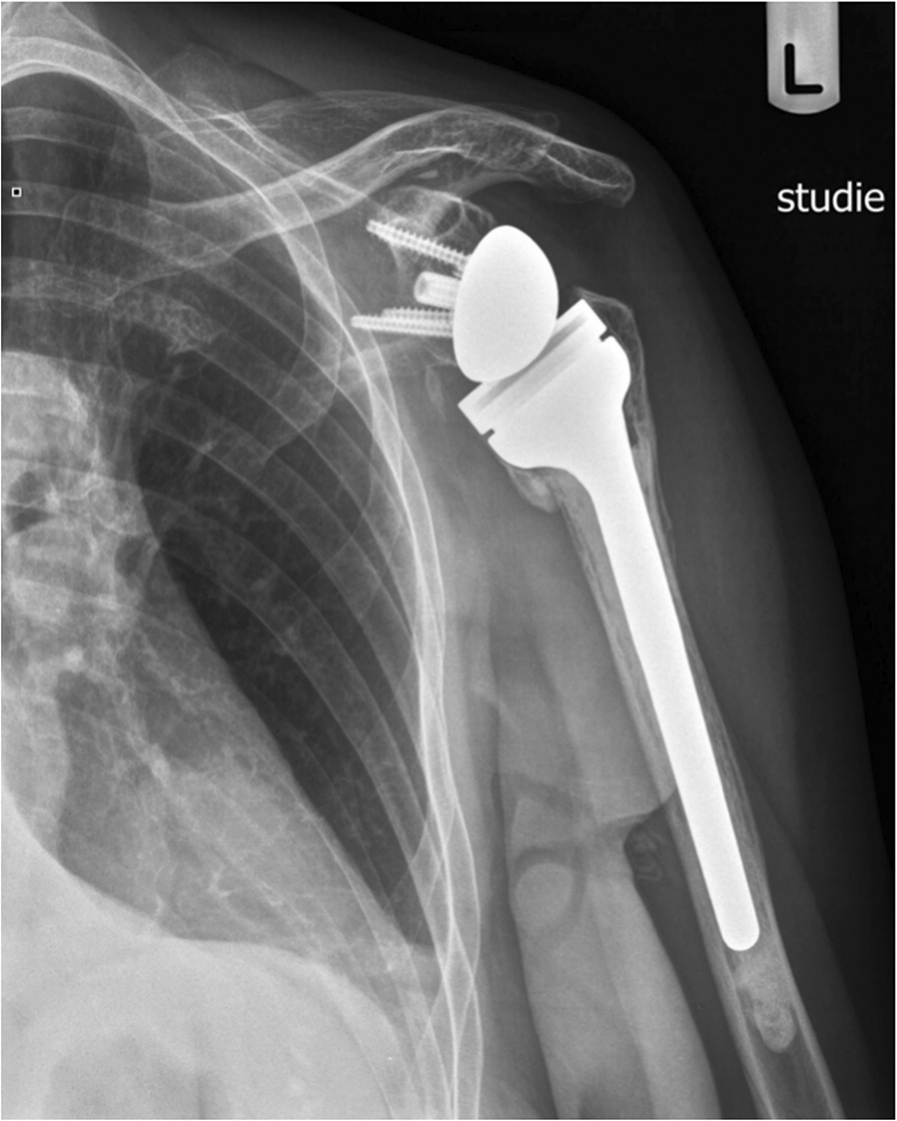
X-ray one year after implantation of reverse shoulder arthroplasty with healed greater tuberosity (a.-p.-view).

**Figure 13:**
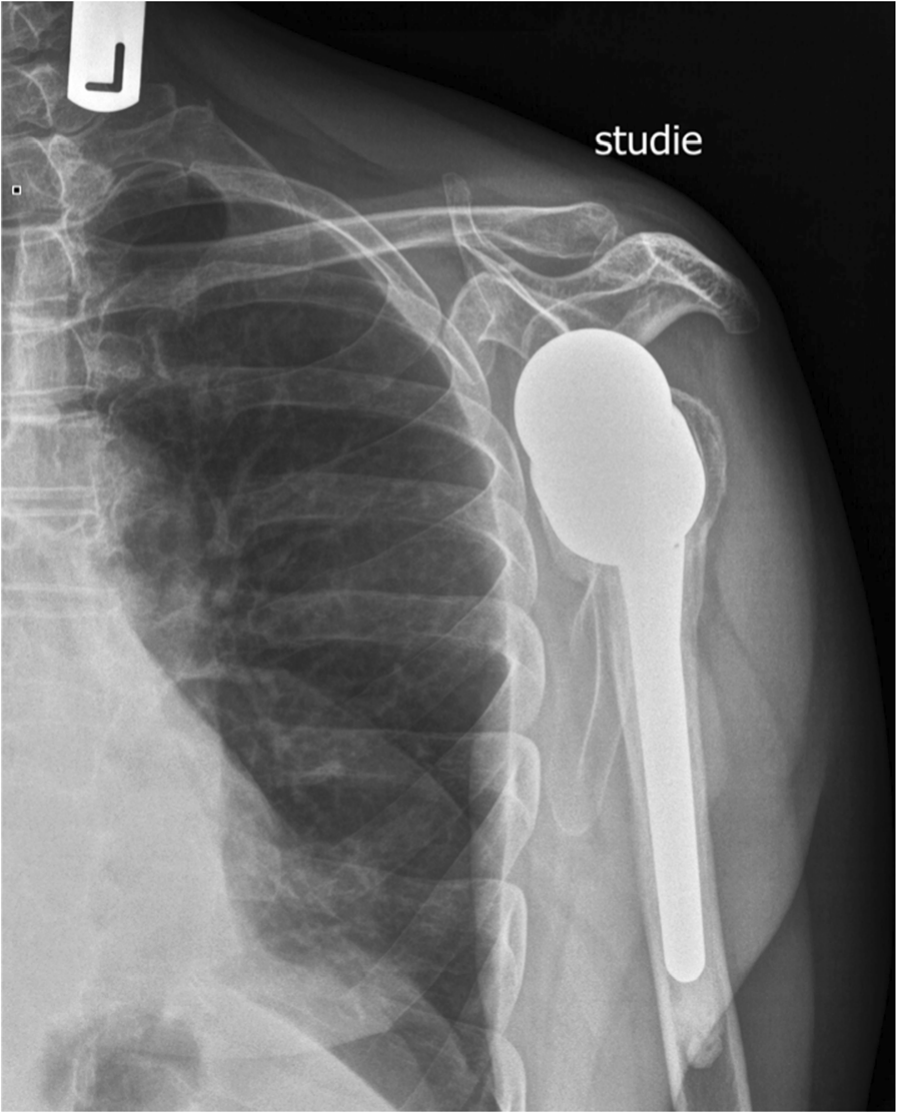
X-ray one year after implantation of reverse shoulder arthroplasty (Y-view).

A displacement of the tuberosities was not noted (Figures 12 and 13). Partial resorption was seen in 8 (50%) cases. One patient had a full resorption of the tubercle.

We encountered 7 cases with stage 1 scapular notching. It did not influence the function or pain in any of these patients. We did not observe any heterotopic ossification in our study.

## Discussion

The treatment of complex 3- and 4-part fractures of the proximal humerus with high risk of avascular necrosis represents a difficult problem for the surgeon. In our experience and according to the literature, methods such as plate or nail fixation may have a significant complication rate and unpredictable functional outcome.

Our mid-term follow-up shows satisfying results for the treatment of severe displaced fractures in elderly patients using RSA. According to our results, RSA can provide immediate relief and good shoulder function for elderly patients with severe PHF.

Nevertheless, the question of longevity of these implants remains to be observed. Long term follow-up studies for RSA as the primary treatment of PHF are necessary in order to assess the incidence of late or long-term complications with respect to component longevity, loosening, duration of pain relief and reduction of strength and ROM.

In a study by Favard et al. [9] the authors found in a population with a mean age of 73 years a decrease in the relative CMS from 88% at under five years to 78% after more than 9 years. Additionally, the authors found that 72% of the patients had a CMS of less than 30 (defined as an end-point) after 10 years. In another study by Guery et al. [10] a survivorship of 58% after 10 years is reported.

In patients younger than 65 years improved function is reported to be maintained for up to 10 years [11].

Patients with severely displaced 3- or 4-part proximal humerus fractures are at high risk of suffering avascular necrosis of the humeral head [12].

Open reduction and internal fixation (ORIF) is often difficult to achieve, as fragment displacement, comminution and osteoporotic bone quality act as limiting factors.

For these reasons, humeral head replacement is indicated when ORIF is not possible, or in cases where there is a high risk of avascular necrosis of the humeral head according to Hertel’s studies [7,13,14].

Satisfactory shoulder function after hemiarthroplasty can be inadequate due to the problem of tuberosity fixation and preexisting rotator cuff disease with or without arthopathy especially in older patients. Poor shoulder function after hemiarthroplasty is often associated with nonunion, displacement and resorption of tuberosity fragments, which often leads to revision arthroplasty using an inverse design [15].

While hemiarthroplasty has been seen to produce satisfactory pain control there are problems with limited range of motion and shoulder function when it is used as a treatment for severe proximal humerus fractures [4,16].

In a study by Boons et al. [17] the authors investigated in a randomized controlled trial the outcome after hemiarthroplasty was used to treat four-part fractures in patients older than 60 years. In 25 patients treated with hemiarthroplasty they established a mean CMS of 64, a mean abduction of 77° and anterior flexion of 98°. In two patients they found postoperative tuberosity resorption. However, they noted that there may have been more disrupted tuberosities which were not observed on plain radiographs.

Comparative literature contrasting the functional outcome of RSA to hemiarthroplasty in the management of PHF is relatively limited. However, functional outcomes showed no significant difference between these two methods and were even higher in the RSA group according to CMS [18,19].

In a comparative study by Garrigues et al. [20] the authors investigated 23 patients of whom 12 had undergone hemiarthroplasty and 11 had undergone reverse total shoulder arthroplasty for proximal humeral fractures in elderly patients. The mean ASES score of the RSA group was 81.1 (range 75-88) and was significantly better than the hemiarthroplasty group with a score of 47.4 points (range 30-81) (p < 0.05). The mean forward elevation was 121° (range 90°-145°) for the RSA group and 91° (range 30-140°) for the hemiarthroplasty group, respectively (p < 0.05).

In our study, results for RSA compare favourably with than the functional outcome for hemiarthroplasty in such patients. Our own results are encouraging and correlate with previously published reports from other trauma centers.

Proximal humeral head fractures often occur in low-demand patients, who can have multiple comorbidities and reduced requirements with regard to shoulder function. In this population group, the primary goal of treatment is pain-free shoulder mobility that provides good function for daily activities and personal hygiene requirements.

Use of primary RSA in the treatment of complex 3- and 4-part fractures of the humeral head remains controversial in Austria. It was previously not routinely performed in our trauma center. There are also certain limitations associated with RSA - the age and the comorbidities of the patients, for example, must of course always be taken into account.

Since 2008, after experiencing unsatisfying functional outcomes for treatment with hemiarthroplasty or plate fixation, we decided to treat patients over 65 years of age or low-demand patients with complex 3- and 4-part PHF and high risk for avascular necrosis and/or with preexisting comorbidities with primary RSA. Our preferred form of treatment would otherwise be ORIF using a plate or alternative methods such as humerus-block in selected cases.

As we have only operated on the worst forms of fractures in elderly or low-demand patients, we achieved a relatively satisfying functional outcome score of 54.8 in CMS, which is comparable to those described in the published literature [4,12,18,21].

We attempted to repair the tuberosities in all cases as there is evidence that healing of the tuberosities is associated with better functional outcome [5,22-24]. Satisfactory outcome also depends on the positioning of the glenosphere in the lower part of the glenoid in order to prevent scapular notching [21,25,26].

The difference in the functional outcome by comparing the ROM, the grip strength and the pain relief in the non-affected and the affected shoulder did not have a major influence on day-to-day activities of the patients as the CMS and the DASH scores showed good results. Our results for pain reduction and range of motion correlate with results observed in previously published literature for this procedure [10,21,27,28].

We observed good functional results during rehabilitation with an early start of passive and active motion although a sling fixation was used for 4-6 weeks. This can also be associated with improved acceptance of the implant by the patient as RSA provides immediate stability of the shoulder.

We encountered some system related complications including a case of transient axillary nerve impairment, which resolved completely after 1 month without additional therapy. A case of dislocation of the shoulder lead to revision with a thicker inlay. The patient did not experience any further instability of the shoulder. We also observed 2 cases of infection. One was a superficial infection that was treated with debridement and did not lead to further complications. The other was a deep infection related to the prosthesis that led to explantation of the prosthesis in a multimorbid patient.

The study has several limitations. Firstly, not all of our operated patients fulfilled the inclusion criteria and we therefore had a highly selected group of patients. Secondly, the average follow up time of 20 months is relatively short. Thirdly, there was a lack of comparison group for comparison of results for ORIF or hemiarthroplasty for example.

## Conclusion

Our mid-term follow-up shows satisfying results for the treatment of severe displaced fractures in elderly patients using RSA. RSA can provide immediate relief and good shoulder function in elderly patients. Nevertheless, the question of longevity of these implants remains to be observed.

## Competing interests

The authors declare that they have no competing interests.

## Authors’ contributions

GM was responsible for conception, design, acquisition of data, analysis and interpretation of data and drafting the manuscript. LM has been involved in drafting the manuscript and interpretation of data. RO has been involved in critical revision of the manuscript and has improved the discussion section regarding complications associated with RSA. PW participated in the design of the study and was responsible for clinical management of the patients. RK has been involved in critical revision of the manuscript with regard to important intellectual content. AK provided general supervision of the research group and was involved in critical revision of the manuscript. All authors read and approved the final manuscript.

## Pre-publication history

The pre-publication history for this paper can be accessed here:

http://www.biomedcentral.com/1471-2474/14/231/prepub
